# Post-surgical staphylococcal toxic shock syndrome in pediatrics: A case report

**DOI:** 10.1016/j.ijscr.2021.106587

**Published:** 2021-11-10

**Authors:** Yousef S. Abuzneid, Abdelrahman Rabee, Hussam I.A. Alzeerelhouseini, Deema W.S. Ghattass, Nermeen Shiebat, Radwan Abukarsh

**Affiliations:** a Al-Quds University, Faculty of Medicine, Jerusalem, State of Palestine; b Palestine Red Crescent Society Hospital, Hebron, State of Palestine

**Keywords:** Post-surgical, Infection, Toxic shock syndrome, Pediatric surgery

## Abstract

**Introduction:**

Toxic shock syndrome (TSS) is a rare but serious, life-threatening medical condition and potentially lethal if not detected and treated early. It is mainly caused by a toxin called toxin-1 produced by *Staphylococcus aureus*, and characterized by fever, hypotension, rash, skin desquamation and multisystem involvement.

**Case presentation:**

Herein, we describe a nine-month-old male patient who presented to the hospital complaining of fever, vomiting and hypoactivity on day one post-orchidopexy. During hospitalization, his condition began to deteriorate with signs and symptoms of multisystemic failure. Laboratory tests and radiological images were done, leading to the decision to reopen and drain the surgical wound. Wound and nasal swabs were cultured and showed *S. aureus* infection, and the diagnosis of toxic shock syndrome was confirmed.

**Discussion:**

TSS is a systemic illness resulting from overwhelming host response to bacterial exotoxins, that cause T cells activation and the release of pro-inflammatory cytokines (IL-1 and TNF-α causing fever, hypotension, and tissue injury). Also, it can present with CNS signs that may be misdiagnosed with meningitis in pediatrics.

It requires early identification and treatment despite its rarity with mortality rate of 81% even with treatment.

The patient's presentation, examination and laboratories tests with the blood and wound cultures were highly suggestive for this condition.

**Conclusion:**

Physicians must maintain a high index of suspicion for TSS, as early diagnosis and treatment make a difference. This condition shouldn't be excluded even in young age patients or after simple procedure as in our case in which TSS occurred after orchidopexy.

## Highlights

• Toxic shock syndrome is a life-threatening condition that can be highly mortal even with treatment.
• Post-surgical TSS is very rare and according to the literature this is the first reported case post-orchidopexy.
• Broad spectrum antibiotics with debridement of the necrotic tissue as management are the best option to treat this condition.

## 1 Introduction

Toxic shock syndrome (TSS) is a toxin-mediated, acute, and potentially life-threatening condition that is usually precipitated by infection with either *Staphylococcus aureus* or group A *Streptococcus* (GAS), also called *Streptococcus pyogenes* [1]. And it mainly results from an overwhelming host response to bacterial superantigens that activates T lymphocytes with a massive release of proinflammatory cytokines responsible for fever, rash, septic shock, and multiple organ failures [2]. The mortality in pediatric patients approaches 5% for TSS secondary to *S. aureus* and up to 10% for TSS secondary to *Streptococcus pyogenes* [3].

Postoperative TSS is a rare clinical entity occurring in only 0.003% of surgical cases [4]. Herein, we present a rare and interesting case of postoperative Staphylococcal TSS in a nine-month-old male which is, according to our literature, the first reported case of TSS occurring after orchidopexy.

The work has been reported in line with the SCARE 2020 criteria [17].

## 2 Case presentation

A nine-month male infant came to our ER (*Palestine Red Crescent Society Hospital*, *Hebron*, *State of Palestine*) complaining of high-grade fever with vomiting and hypoactivity after one day post-operation due to a left non-palpable undescended testis. The fever was registered as 39 °C axillary and the patient also manifested irritability and was found to be in severe dehydration, so he was admitted as a case of viral gastritis in another hospital.

During the course of his hospitalization (thirteen days), he also developed watery diarrhea which was associated with poor feeding and abdominal distention. Moreover, his condition continued deteriorating, showing signs and symptoms of abdominal distention, rigidity, tenderness, high grade fever, decreased urine output, drowsiness, and bloody stool. After that, the infant was referred to our hospital for further investigations and management.

On arrival, laboratory tests and a detailed physical examination were performed. The patient looked ill, tachypneic and grunting with a blood pressure of 90/40 mmHg, abdominal distention, rigid abdomen (more on the left side where a left inguinal incision of 5 cm was present) and tenderness. He also was hypoactive and semiconscious with a GCS (Glasgow Coma Scale) of 8 [18].

Laboratory findings showed that he had a low hemoglobin level of 7.5 g/dL, a high WBCs count of 15.5 × 10^3^/μL and a low platelet count of 54 × 10^3^/μL. He also had low potassium (2.7 mmol/L), high urea (33 mg/dL), high ammonia (80.5 μ/dL), high AST (192 U/L) and ALT (739 U/L), high PT (33 s) and INR (2.32) and low albumin (2 g/dL).

Blood gases also showed metabolic acidosis with compensatory respiratory alkalosis in which the pH was 7.35 with a pCO_2_ level of 16 mmHg and a HCO_3_ level of 9 mEq/L.

After few hours, the patient developed O_2_ desaturation, so he was connected to oxygen by nasal cannula (0.5–1 L) and transferred to the Pediatric Intensive Care Unit (PICU) in our hospital. Consequently, two peripheral cannulas were inserted, and blood cultures were taken. We also kept the patient NPO (*nil* per os) and gave an IV (intravenous) fluid expansion with dextrose saline (DS) 0.45 with 7% dehydration and maintenance and started him on meropenem (200 mg once every 8 h) and vancomycin (200 mg once every 8 h).

Due to his low platelets levels and abnormal coagulation profile, packed RBCs, fresh frozen plasma (FPP) and vitamin K were given.

**Fig. 1:**
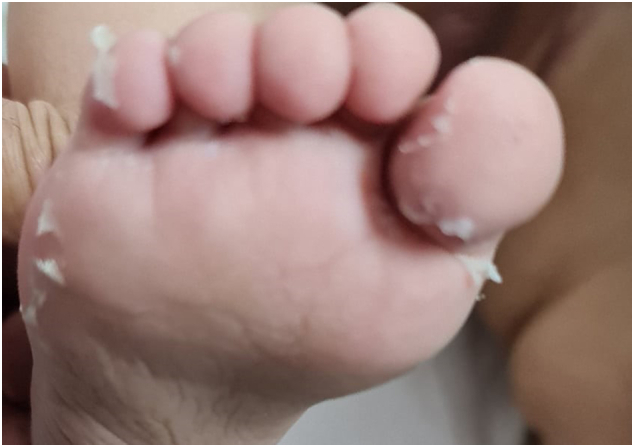
Skin desquamation seen in post-operative day 10.

An abdominal ultrasound was executed and showed dilated aperistaltic bowel loops but with good vascularity and no intussusceptions or volvulus. It also showed hepatomegaly (10 cm). More radiological images were performed, and an abdominal and chest CT scan showed a congested groin in which the left inguinal as well as the left abdominal wall were extending to the lower chest fatty plane containing few air bubbles, suggesting fasciitis with turbid fluid. Diffuse bowel loop dilation mostly due to an ileus and an enlarged liver of 10 cm were also noticed and there was evidence of bilateral pulmonary lower lobe infiltrates with ground glass appearance and/or atelectasis. Skin desquamation on both feet was seen on post-operative day 10 (Fig. 1).

Afterwards, the surgical wound was opened, which secreted dirty fluid and made another incision over the most indurated area which also secreted dirty fluid. We kept the wound and the incision opened with drains on them and took a wound swab for culture which, later on, showed *Staphylococcus aureus* infection. We also took a nasal swab and blood and urine cultures giving as positive results only in the nasal swab for *Staphylococcus aureus* as well.

After an antibiotic sensitivity test for the bacteria, we started the patient on clindamycin and tazocin.

NPS (nasopharyngeal swab) for COVID-19 was also taken and was positive, so the patient was started also on azithromycin and budecort nebulizer.

On the second day of admission, a central line was inserted under US guidance and the patient was sent to the OR (operating room) where we did a surgical debridement of the wound under general anesthesia because there was infected necrotic tissue in the left inguinal region extending to the loin but with a healthy fascia. During the surgery, a second swab culture from the wound was taken and showed no bacterial growth.

**Fig. 2:**
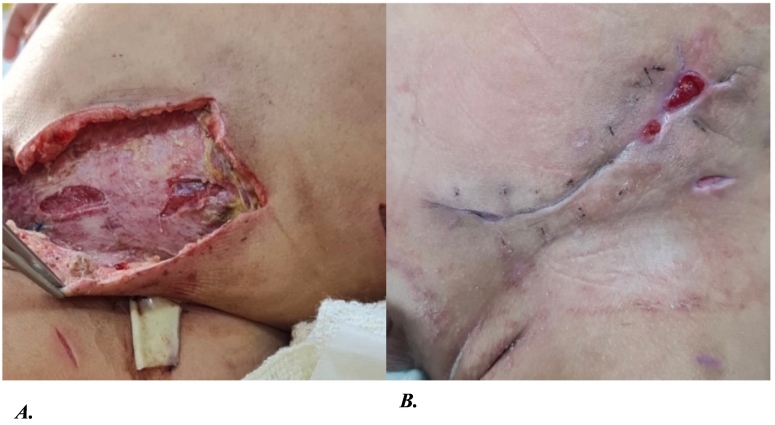
A. Opened wound for drainage after debridement of infected necrotic tissue. B. Closed wound after the removal of the drainage showing the skin in good conditions with no discharge before going home.

After the surgery, the patient had an endotracheal tube (ETT) and was connected to a mechanical ventilator under sedation for four days, remaining hemodynamically stable and weaning gradually the sedation until extubation. Meanwhile, there was daily wound care and dressing (Fig. 2) until the wound became healthy and without discharge so we could close the wound over a drain.

We started to feed him gradually until well tolerated and full feeding capacities once he was extubated and awake. After one-week post-operation, the patient was discharged home in good general conditions.

## 3 Discussion

Toxic shock syndrome (TSS) is a systematic severe illness which is characterized by shock, pyrexia, gastrointestinal disturbances, an erythematous rash, and central nervous system signs including lethargy or irritability (which can be misdiagnosed with those signs of meningitis in pediatrics) [5].

Streptococcus and staphylococcus bacteria can both cause this condition through the elaboration of toxins. *Streptococcus pyogenes* (specially the M1 and M3 serotypes) and *Staphylococcus aureus* are the most common microorganisms associated with TSS [6].

These organisms cause an overwhelming host response to bacterial superantigens through mitogenic exotoxins which bind to a major histocompatibility complex class-II (MHC-2), CD28 costimulatory receptors, and T-cell receptors which furthermore result in a constitutive activation of T-cells and proinflammatory cytokine release [7].

Different strains of *Staphylococcus aureus* produce TSS toxin 1 and staphylococcal enterotoxin B and C, products that act as superantigens. These superantigens bind and activate T-cells indiscriminately, leading to the amplification of cytokines TNF-α and IL-1 (together, causing fever, hypotension, and tissue injury) [8].

Postoperative TSS is extremely rare but going through the literature, it has been documented as a complication from a broad range of procedures. A contaminated product (i.e., napkin and tampon) as a cause of postoperative staphylococcal TSS was identified only in very few cases [9].

As mentioned in the introduction, only 0.003% of surgical cases has been identified as postoperative TSS [4].

As we can see in our case and as mentioned in the case presentation, our patient came complaining of high-grade fever, vomiting, hypoactivity, with a picture of gastritis and severe dehydration, which progressed to other signs and symptoms of shock. His laboratory results also indicated shock and the patient was deterioration fast, so we started the protocol for septic shock and gave the patient IV fluids and broad-spectrum of antibiotics as an initial treatment.

However, it was not until the culture results and the physical manifestations of this condition (like the skin desquamation and the secretion of dirty fluid from the incision of the previous operation) that we realized the diagnosis.

This condition can be listed with other differential diagnosis such as Kawasaki disease, scarlet fever, Rocky Mountain Spotted fever and leptospirosis [13].

Moreover, untreated TSS is fatal and mortality rates up to 81% have been reported even if the patients were treated. Herein, cases of TSS require a high index of suspicion, early antibiotic administration, and aggressive hemodynamic support [10] since hemodynamic instability and cardiovascular collapse are terminal manifestations of this condition and need treatment with aggressive fluid resuscitation and vasopressor support [6].

Broad-spectrum antibiotics for the coverage of MRSA and streptococcus (i.e., vancomycin and linezolid) and gram-negative bacteria need to be initiated until antibiotic sensitivity lab results come (then switch to the appropriate antibiotic). Also, clindamycin should be used to counteract the systemic effects that the toxins will produce after their release [6].

Therapy with corticosteroids for TSS is poorly studied, with few papers demonstrating a reduction in the duration of fever and severity of the illness [11], [12].

The administration of intravenous immune-globulin or also known as IVIG (usually 1–2 g/kg) has been proposed in many articles as a potential adjunct in the treatment of TSS due to the effect of immunoglobulins by blocking the activation of T-cells by both staphylococcal and streptococcal superantigens. They also contribute by improving bacterial opsonization, phagocytosis, and destruction [14], [15], [16].

In comparison to the case that we present, we indeed started with broad-spectrum antibiotics which were vancomycin and meropenem. However, we did not include corticosteroids nor IVIG to his treatment. Also, in was not until we received the laboratory and culture results about the incision that we diagnosed him and switched to more specific antibiotics (clindamycin and tazosin) after the sensitivity tests results were concluded.

We also had to re-open the incision, debride the necrotic tissue and let it open for wound care and dressing to a better control and better care, preventing another future complication.

After full recovery and good food intake, we were able to discharge the patient to his house in good conditions.

To summarize, the infant that we present in this case report was referred to our hospital as a case of gastritis with severe dehydration. Thorough examination and laboratory testing, the patient was found to have high grade fever, hypotension and signs and symptoms of multisystemic involvement including diarrhea, decreased urine output, drowsiness, semi-consciousness, and bloody stool and desquamation of the skin. Thus, rising the suspicion for having TSS.

He had low platelets, low hemoglobin, high urea, high ammonia, high liver enzymes and PT and INR, and lot albumin. In addition, wound culture was done and returned positive for *Staphylococcus aureus*, making the diagnosis towards TSS clearer.

Herein, we started his treatment with broad spectrum antibiotics, which we switched to a more specific one after the antibiotic sensitivity test came back.

This case report reflects and fulfils all the criteria for the diagnosis of TSS due to the correlating findings that we mention above.

## 4 Conclusion

We describe a case of a nine-month-old infant who presented to our hospital as a picture of postoperative TSS.

We believe that this reported case (TSS post-orchidopexy) is one of its kind according to the literature review and it should be written so all the physicians can know about it and be aware.

The diagnosis was challenging perse, but full examination and laboratory tests were highly suggestive for the diagnosis. Blood and wound cultures were taken, and the patient was started on broad spectrum antibiotics which then were switched to more specific ones according to the antibiotic sensitivity tests.

Surgical debridement of the wound was performed because due to this condition, necrosis of the skin was notices. After that, the patient was discharged in good conditions a week later.

It is very important that the physicians, especially the surgeons, know about this possible complication after post-orchidopexy because such condition, which is rare but fatal, can be well controlled and easily treated if discovered promptly.

## Ethical approval

The study is exempt from ethical approval in our institution.

## Sources of funding

This research did not receive any specific grant from funding agencies in the public, commercial, or not-for-profit sectors.

## CRediT authorship contribution statement

Study concept or design: Radwan Abukarsh.

Data collection and data analysis: Yousef S. Abuzneid.

Writing the manuscript: Yousef S. Abuzneid, Abdelrahman Rabee, Hussam I. A. Alzeerelhouseini, Deema W. S. Ghattass, Raed A. H. Jubran, Nermeen Shiebat.

Review & editing the manuscript: Yousef S. Abuzneid, Abdelrahman Rabee.

## Registration of research studies

Not applicable.

## Guarantor

Dr. Yousef S. Abuzneid.

## Consent

Written informed consent was obtained from the patient's parents for publication of this case report and accompanying images. A copy of the written consent is available for review by the Editor-in-Chief of this journal on request.

## Declaration of competing interest

There is no conflict of interest.

## References

[1] Silversides J.A., Lappin E., Ferguson A.J. (2010). Staphylococcal toxic shock syndrome: mechanisms and management. Curr. Infect. Dis. Rep..

[2] Ladhani S. (2003). Understanding the mechanism of action of the exfoliative toxins of Staphylococcus aureus. FEMS Immunol. Med. Microbiol..

[3] Chuang Y.Y., Huang Y.C., Lin T.Y. (2005). Toxic shock syndrome in children: epidemiology, pathogenesis, and management. Paediatr. Drugs.

[4] Graham D.R., O'Brien M., Hayes J.M., Raab M.G. (1995). Postoperative toxic shock syndrome. Clin. Infect. Dis..

[5] Slingluff C.L., Burns W.W., Cooperberg C. (1990 Oct). Toxic shock syndrome after inguinal hernia repair. Report of a case with patient survival. Am. Surg..

[6] Chandra R., Gold S., Kohler C., Valladares J., Hennessy S.A., Bhat S.G. (2021 Feb). When all Else fails: a rare case of postoperative toxic shock syndrome arising from surgical site infection after decompressive neurectomy successfully treated with angiotensin-2. Case Rep. Crit. Care.

[7] Proft T., Fraser J.D., Ferretti J.J., Stevens D.L., Fischetti V.A. (2016 Feb 10). Streptococcus pyogenes: Basic Biology to Clinical Manifestations [Internet].

[8] Schlievert P.M., Bohach G.A., Ohlendorf D.H., Stauffacher C.V., Leung D.Y., Murray D.L., Prasad G.S., Earhart C.A., Jablonski L.M., Hoffmann M.L., Chi Y.I. (1995 Nov). Molecular structure of staphylococcus and streptococcus superantigens. J. Clin. Immunol..

[9] Bartlett P., Reingold A.L., Graham D.R., Dan B.B., Selinger D.S., Tank G.W., Wichterman K.A. (1982 Mar 12). Toxic shock syndrome associated with surgical wound infections. JAMA.

[10] Davies H.D., McGeer A., Schwartz B., Green K., Cann D., Simor A.E., Low D.E. (1996 Aug 22). Invasive group a streptococcal infections in Ontario, Canada. Ontario group a streptococcal study group. N. Engl. J. Med..

[11] Vergis N., Gorard D.A. (2007 Feb). Toxic shock syndrome responsive to steroids. J. Med. Case Rep..

[12] Todd J.K., Ressman M., Caston S.A., Todd B.H., Wiesenthal A.M. (1984 Dec 28). Corticosteroid therapy for patients with toxic shock syndrome. JAMA.

[13] Raghavendra P., Kharidehal N. (2005). Staphylococcal toxic shock syndrome in a 3-year-old male child. The internet. J. Infect. Dis..

[14] Lappin E., Ferguson A.J. (2009 May). Gram-positive toxic shock syndromes. Lancet Infect. Dis..

[15] Schmitz M., Roux X., Huttner B. (2018). Streptococcal toxic shock syndrome in the intensive care unit. Ann. Intensive Care.

[16] Mouthon L., Kaveri S.V., Spalter S.H., Lacroix-Desmazes S., Lefranc C., Desai R., Kazatchkine M.D. (1996 May). Mechanisms of action of intravenous immune globulin in immune-mediated diseases. Clin. Exp. Immunol..

[17] Agha R.A., Franchi T., Sohrabi C., Mathew G., Kerwan A., SCARE Group (2020). The SCARE 2020 guideline: updating consensus Surgical CAse REport (SCARE) guidelines. Int. J. Surg..

[18] (1993).

